# Relative validity of three diet quality scores derived from the Brief-type Self-administered Diet History Questionnaire and Meal-based Diet History Questionnaire in Japanese adults

**DOI:** 10.1017/S0007114524002058

**Published:** 2024-12-28

**Authors:** Fumi Oono, Kentaro Murakami, Nana Shinozaki, Nana Kimoto, Shizuko Masayasu, Satoshi Sasaki

**Affiliations:** 1 Department of Social and Preventive Epidemiology, Division of Health Sciences and Nursing, Graduate School of Medicine, The University of Tokyo, Tokyo, Japan; 2 Department of Epidemiology and Preventive Medicine, Gifu University Graduate School of Medicine, Gifu, Japan; 3 Department of Social and Preventive Epidemiology, School of Public Health, The University of Tokyo, Tokyo 113-0033, Japan; 4 Ikurien-naka, Ibaraki, Japan

**Keywords:** Quality of diet, Diet quality index, FFQ, Validation, Reliability, Diet surveys, Dietary patterns, Dietary Approaches to Stop Hypertension, Diet, Mediterranean, Japanese

## Abstract

No study has validated questionnaires for assessing easily calculable diet quality scores in Japan. The Brief-type self-administered Diet History Questionnaire (BDHQ) is widely used to assess dietary intake in Japan, while the Meal-based Diet History Questionnaire (MDHQ) assesses dietary intake for each meal (breakfast, lunch, dinner and snacks) and overall dietary intake. This study examined the relative validity of the BDHQ and MDHQ for assessing three diet quality scores in Japanese adults. A total of 111 women and 111 men aged 30–76 years completed the web MDHQ and BDHQ, followed by 4-non-consective-day weighed dietary records. The diet quality scores examined included the Diet Quality Score for Japanese (DQSJ), Dietary Approaches to Stop Hypertension (DASH) score and Alternate Mediterranean Diet (AMED) score. The means of the three scores for overall diet from the BDHQ were not significantly different from those from the dietary records in both sexes, whereas those from the MDHQ were higher than those from the dietary records, except for the DASH and AMED in women. Pearson’s correlation coefficients between both questionnaires and dietary records were 0·57–0·63 for DQSJ, 0·49–0·57 for DASH and 0·31–0·49 for AMED across both sexes and both questionnaires. For each meal, Pearson’s correlation coefficients between the MDHQ and dietary records ranged from 0·01 (DASH for snacks in women) to 0·55 (DQSJ for breakfast in men), with a median of 0·35. This study showed that the ability of the BDHQ and MDHQ to rank individuals was good for DQSJ and DASH and acceptable for AMED.

In recent decades, nutritional epidemiological research has increasingly focused on overall dietary intake rather than the intake of specific foods or nutrients^(1)^. One major approach to evaluating overall dietary intake is diet quality score, a summary score of dietary intake based on predefined criteria^(2,3)^. Among various scores, the Dietary Approaches to Stop Hypertension (DASH) scores^(4)^ and Mediterranean diet score^(5)^ are two of the most used scores worldwide. These scores are not complex, allowing researchers across the globe to apply these scores to various populations. A large body of evidence has shown that the two scores have protective associations with mortality and non-communicable diseases such as CVD and type 2 diabetes mellitus in various populations, but most evidence has come from Western countries^(6–8)^.

Compared with Western populations, the Japanese population has markedly different intakes of some food groups and nutrients, such as higher intakes of seafoods and Na and lower intakes of whole grains, nuts and added sugars^(9,10)^. When assessing diet quality, it is essential to consider dietary intake within the population^(11,12)^. Therefore, we recently developed the Diet Quality Score for Japanese (DQSJ) on the basis of dietary intake in Japan and existing diet quality scores which have established their associations with health outcomes mainly in Western countries, including the DASH and Mediterranean diet scores^(13)^. The DQSJ showed favourable associations with intakes of most nutrients in Japanese adults^(13)^, but its associations with health outcomes have not been examined. Additionally, only a few studies have examined whether DASH and Mediterranean diet scores also have protective associations with health outcomes in Japan^(14–17)^, possibly due to a lack of validated assessment tools.

Examination of associations between diet quality and health outcomes requires valid instruments for assessing diet quality. The validity of diet quality scores derived from dietary assessment questionnaires has been evaluated in several countries^(18–23)^. Previous Japanese studies have examined the relative validity of questionnaires for estimating the Healthy Eating Index (HEI)-2015 and the Nutrient Rich Food Index 9.3 (NRF9.3)^(19,20)^. However, calculating HEI-2015 and NRF9.3 needs a specific database (e.g. a food composition database for added sugar and a database for weight conversions to the specific units of the HEI-2015), resulting in limited availability of these scores. No study has examined the validity of easily calculable diet quality scores derived from questionnaires in Japan. The DQSJ, DASH and Mediterranean diet score are easily calculated without a specific database. Therefore, validated questionnaires for assessing these scores may promote the conduct of future studies about diet quality in Japan.

A Brief-type Self-administered Diet History Questionnaire (BDHQ) is one of the most widely used dietary assessment questionnaires in Japan, as evidenced by approximately 550 citations of validation studies of the BDHQ^(24,25)^ according to the Web of Science (on 10 January 2024). The BDHQ has been used for many purposes^(26)^, including interventions for diet^(27)^, administrative surveys^(28)^ and epidemiological studies for adults^(29)^, young adults^(30)^, elderly individuals^(31)^, pregnant women^(32)^ and individuals with diseases^(33)^. Establishing the validity of diet quality scores derived from the BDHQ will further promote the examination of associations between diet quality and various health outcomes in diverse populations in Japan. Additionally, while most existing dietary questionnaires, including BDHQ, cannot assess meal-specific intake, a novel Meal-based Diet History Questionnaire (MDHQ) was recently developed to assess dietary intake of each meal type (breakfast, lunch, dinner and snacks)^(34–36)^. A validated questionnaire for each meal will promote future research on chrono-nutrition, such as the relationship between diet quality of each meal and health outcomes^(37,38)^.

Therefore, the aim of this study was to examine the relative validity of the three diet quality scores (DQSJ, DASH and Mediterranean diet score) for the overall diet derived from the BDHQ and MDHQ and for each meal derived from the MDHQ against dietary records. We examined the validity of the web versions of the BDHQ and MDHQ as a primary aim and the validity of their paper versions as a secondary aim. The validity was examined by stratifying by sex, as this kind of information is essential for future research in which the dietary assessment questionnaires are employed in only male or female participants.

## Methods

### Study design and participants

This validation study was based on data from a survey conducted between August and October 2021 in fourteen of forty-seven prefectures in Japan. The details of the survey have been described elsewhere^(20,35,36)^. In brief, sixty-six research dietitians recruited participants using a convenience sampling method. To maximise the completion rate, research dietitians recruited those who had a full understanding of the study procedure and were willing to complete the entire survey. For each prefecture and each of the four age categories (30–39, 40–49, 50–59 and 60–69 years), two healthy women were recruited with their husbands (irrespective of the husbands’ age), resulting in a total of 112 women and 112 men invited. This sample size was determined based on the recommendation of Cade and colleagues that 100 or more participants are desirable for a validation study^(39)^. Exclusion criteria of the potential participants were dietitians, those living with a dietitian, those who received dietary counselling from a doctor or dietitian, those with diabetes or receiving insulin therapy, those receiving dialysis treatment, those with difficulty completing the web-based questionnaire, and pregnant or lactating women. These exclusion criteria were developed to examine the validation of dietary assessment questionnaires in the general population. The number of invitees excluded from the survey was not recorded due to the recruiting strategy used.

Participants were asked to complete web questionnaires comprised of the MDHQ (web MDHQ), the BDHQ (web BDHQ) and questions about basic characteristics (e.g. smoking and education status). Starting 7–10 d after the web questionnaire response, participants completed a 4-non-consecutive-day weighed dietary records within 2 weeks. Finally, participants completed the paper versions of the MDHQ and BDHQ, from after the day of their last dietary records. Each couple received a voucher worth 5000 Japanese yen (equivalent to 35 US$ as of 1 July 2023) after the completion of the study.

The study was conducted in accordance with the guidelines of the Declaration of Helsinki. The study protocol was approved by the committee of the University of Tokyo, Faculty of Medicine (protocol code. 2020326NI). The study purpose and protocol were explained before the study, and written informed consent was obtained from all participants.

### Dietary questionnaires

The BDHQ is a self-administrated structured questionnaire which enquires about the consumption frequency of selected foods commonly consumed in Japan, general dietary behaviour, and usual cooking methods in the previous month^(24,25)^. Daily intake of foods (fifty-eight food items in total), energy and selected nutrients was calculated using an *ad hoc* computer algorithm for the BDHQ. The intake of all participants was calculated based on a sex-specific fixed portion size derived from recipe books for Japanese dishes, rather than through the collection of portion size information^(24,25)^.

The MDHQ is a self-administered structured questionnaire designed to estimate intakes of energy, nutrient and food in the previous month for each meal type (breakfast, morning snack, lunch, afternoon snack, dinner and night snack)^(34)^. The MDHQ consists of three parts. The first part asks about the consumption frequency of generic food groups (*n* 11–24) for each meal type, with options from 0 to 7 d per week. The second part asks about the relative consumption frequency of sub-food groups within generic food groups regardless of meal type, with options of ‘always’, ‘often’, ‘sometimes’, ‘rarely’ or ‘never’. The information of the first and second parts are used to estimate a large number of foods effectively based on a limited number of questions. The third part asks about general eating behaviours including usual cooking methods. The dietary intake of all participants was calculated based on a sex- and meal type-specific fixed portion size determined using dietary records data of 242 Japanese adults, except for alcohol intake, for which portion size was directly asked^(34)^.

The web BDHQ and web MDHQ were identical to their paper versions in terms of content. The web version of both questionnaires was created using Google Forms and did not allow for a non-response to any question. In the paper versions of the questionnaires, the responses to all questions were checked by the research dietitians and the research centre staff, who asked participants to re-answer missing or multiple answers for a single-choice question. The diet quality scores were calculated using dietary intake derived from the web and paper versions of the BDHQ and MDHQ.

### Dietary record

A 4-non-consecutive-day weighed dietary records, used as a reference method, consisted of three working days and one non-working day. Each couple was provided with a digital scale (KS-274, Dretec), recording sheets and a manual for the dietary records. The research dietitians explained the recording method verbally and in writing. Participants were asked to weigh and record all consumed food and drink, both in and out of the home. When weighing was difficult (e.g. eating out), they were asked to document as much information as possible, such as the brand name of the food, the consumed portion size and the details of leftovers. The recording sheets for each record day were submitted directly to the research dietitian after recording. Research dietitians then reviewed these sheets and obtained additional information via phone, email, message app or in-person interview when necessary. The research dietitians and trained staff at the central office of the survey reconfirmed all collected records. Individual food items were coded, and intakes of energy and nutrients were calculated, based on the Standard Tables of Food Composition in Japan, 2015^(40)^. The dry weight of whole grains, such as brown rice before cooking, buckwheat flour in buckwheat noodles and whole grain flour in bread, was estimated using information concerning ingredients and weight change on cooking in the Standard Tables of Food Composition in Japan^(40)^. Dietary supplements were not used in the calculation of dietary intake. Diet quality scores were calculated by averaging the 4-d intake of the entire day and of each meal type (breakfast, lunch, dinner and sum of all snacks).

### Calculation of diet quality scores

Three diet quality scores were calculated: the DQSJ^(13)^, DASH score^(41)^ and Alternate Mediterranean Diet (AMED) index ^(42)^. Total possible score ranges are 0–30 for the DQSJ, 8–40 for the DASH and 0–9 for the AMED, with higher scores indicating a higher diet quality. All scores are calculated using intake distribution stratified by sex. The DQSJ consists of ten components, each given 0–3 points based on the quartile of intake of each component in the study population. The highest quartile is assigned three points for seven components (fruits, vegetables, whole grains, dairy products, nuts, legumes, and fish), whereas the lowest quartile is assigned three points for three components (red and processed meat, sugar-sweetened beverages, and sodium). The DASH score comprises eight components, each given 1–5 points based on the quintile of intake of each component in the study population. The highest quintile is assigned five points for five components (fruits and fruit juice, vegetables, whole grains, nuts and legumes, and reduced-fat dairy products), whereas the lowest quintile is assigned five points for three components (red and processed meat, sugar-sweetened beverages, and sodium). The AMED consists of nine components, each given 0 or 1 point based on median intake of each component in the study population except for alcohol. It includes seven favourable components (fruits and fruit juice, vegetables, whole grains, nuts, legumes, fish, and the ratio of monosaturated fatty acids to saturated fatty acids), one unfavourable component (red and processed meat) and one component with an optimal intake range (alcohol). Further details regarding the calculation of each score are described in online Supplementary Tables 1 (for dietary records) and 2 (for the BDHQ and MDHQ). All scores were calculated using energy-adjusted intake of components (per 4184 kJ for the entire day or each meal), except for alcohol intake without energy adjustment (g/d) in the AMED. When participants reported no consumption of energy-providing food and drinks in a meal (defined as non-consumer), their intakes of all components in the meal were set at 0 g/4184 kJ, and they received nine points for the DQSJ, fifteen points for the DASH and one point for the AMED. For snacks assessed using the MDHQ, all participants were assigned no consumption of vegetables whole grains, nuts, legumes, fish, and red and processed meat intake because the MDHQ does not assess these intakes.

### Sociodemographic variables

The MDHQ also includes questions about basic characteristics (i.e. sex, age, body height and body weight). BMI was calculated by dividing body weight (kg) by the square of body height (m). Additionally, the highest level of educational attainment and smoking status were asked about at the end of the web questionnaire. The options for the highest level of educational attainment included junior high school, high school, junior college or technical school, and university or graduate school. These were then categorised into three groups: junior high school or high school, junior college or technical school, and university or higher. Smoking status was categorised as either current smokers or non-smokers (including never-smokers and past smokers who quit more than a year ago), based on participants’ responses.

### Statistical analysis

All analyses were stratified a priori by sex. Descriptive values of variables were presented as mean and standard deviation or percentages. The mean values of the diet quality scores derived from the web BDHQ and web MDHQ were compared with those derived from the dietary records using a paired *t* test to assess estimation ability at the group level. The ability of the two questionnaires to rank individuals in a population was evaluated using Pearson’s correlation coefficients between the two questionnaires and dietary records. Pearson’s correlation coefficients were used because no outliers of diet quality scores existed, and the scores’ distribution was not so skewed when evaluated using histograms. Deattenuated correlation coefficients for day-to-day variation were not calculated because diet quality scores were calculated using the mean dietary intakes for the 4-d dietary records (not each day). To evaluate the agreement and proportional bias of the diet quality scores between the two questionnaires and dietary records, Bland–Altman plot analysis was used with linear regression analysis^(43)^. The 95 % limits of agreement were calculated as the mean (sd 1·96) of the differences between the questionnaire and dietary records^(43)^. In Bland–Altman plots, all scores were transformed into possible score ranges of 0–100 to allow comparison of the limit of agreement between the three scores. Analyses identical to the above were performed for the diet quality scores of each meal assessed by the web MDHQ. The identical analyses were also conducted for the diet quality scores for overall diet derived from the paper versions of the BDHQ and MDHQ. All statistical analyses were performed using SAS statistical software (version 9.4, SAS Institute Inc.), with two-tailed *P* values < 0·05 considered statistically significant.

## Results

This study included 111 men aged 30–76 years and 111 women aged 30–69 years who completed the study protocol. The mean (sd) of age was 52·7 (11·9) years for men and 50·9 (10·7) years for women (Table 1). The mean (sd) of BMI was 23·8 (3·6) kg/m^2^ for men and 22·7 (3·3) kg/m^2^ for women. The proportion of current smokers was 31·5 % for men and 10·8 % for women.

**Table 1.** Basic characteristics of the study population (Mean values and standard deviations; numbers and percentages)

	Men (*n* 111)				Women (*n* 111)			
	Mean	sd	*n*	%	Mean	sd	*n*	%
Age (years)	52·7	11·9			50·9	10·7		
Body height (cm)*	170·2	6·3			158·4	5·4		
Body weight (kg)*	68·9	11·9			56·9	8·5		
BMI (kg/m^2^)^†^	23·8	3·6			22·7	3·3		
Energy intake from dietary records (MJ/d)	9·6	2·1			7·2	1·4		
Energy intake from web BDHQ (MJ/d)	8·5	2·6			6·5	1·7		
Energy intake from web MDHQ (MJ/d)	8·1	2·2			6·2	1·5		
Current smoker			35	31·5			12	10·8
The highest level of educational attainment								
Junior high school or high school			41	36·9			28	25·2
Junior college or technical school			22	19·8			55	49·6
University or higher			48	43·2			28	25·2

BDHQ, brief self-administered diet history questionnaire; MDHQ, Meal-based Diet History Questionnaire.

* Based on self-report.

† Calculated using self-reported body height and weight.

### Scores for overall diet on the web versions of the two questionnaires

The range of energy intake derived from the web BDHQ was 1·8–16·2 MJ/d for men and 2·3–11·5 MJ/d for women, whereas that derived from the web MDHQ was 83·6–16·6 MJ/d for men and 2·5–10·7 MJ/d for women. Table 2 shows the mean and sd values of the total and component scores for the DQSJ, DASH and AMED derived from the dietary records, BDHQ and MDHQ. The mean total scores for the DQSJ, DASH and AMED derived from the BDHQ were not significantly different from those derived from the dietary records in both sexes (*P* = 0·44 for DQSJ, 0·15 for DASH and 0·43, for AMED in men and 0·75, 0·28, and 0·14, respectively, in women). On the other hand, the mean total scores derived from the MDHQ were higher than those derived from the dietary records, except for the DASH and AMED in women (*P* < 0·001 for DQSJ, 0·03 for DASH, and 0·04 for AMED in men and < 0·001, 0·39, and 0·10, respectively, in women). For component scores, the mean scores of whole grains for the DQSJ and DASH were higher in the BDHQ and MDHQ than in the dietary records. In contrast, the mean scores of low-fat dairy products of the DASH were lower in the BDHQ and MDHQ than in the dietary records (except for the BDHQ in men). The mean scores of nuts for the AMED were higher in the MDHQ than dietary records in women but not significant in men (*P* = 0·058). The BDHQ did not include the question about nuts intake, so all participants were scored zero for nuts for the DQSJ and AMED.

**Table 2.** Mean estimates of the diet quality scores derived from the 4-d weighed dietary record, the web version of the Brief-type Self-administered Diet History Questionnaire (BDHQ), and the web version of the Meal-based Diet History Questionnaire (MDHQ) in Japanese adults^†^ (Mean values and standard deviations)

	Men (*n* 111)						Women (*n* 111)					
	Dietary records		BDHQ		MDHQ		Dietary records		BDHQ		MDHQ	
	Mean	sd	Mean	sd	Mean	sd	Mean	sd	Mean	sd	Mean	sd
DQSJ	13·8	5·0	13·6	4·0	15·4**	4·0	13·9	4·7	13·7	4·0	15·5**	4·4
Fruits	1·5	1·1	1·5	1·2	1·5	1·1	1·5	1·1	1·5	1·1	1·5	1·1
Vegetables	1·5	1·1	1·5	1·1	1·5	1·1	1·5	1·1	1·5	1·1	1·5	1·1
Whole grains	0·6	1·0	1·5**	1·1	1·5**	1·1	0·7	1·1	1·5**	1·1	1·5**	1·1
Nuts	0·8	1·1	0·0**	0·0	1·3**	1·2	0·6	1·1	0·0**	0·0	1·5**	1·1
Legume	1·5	1·1	1·5	1·1	1·5	1·1	1·5	1·1	1·5	1·1	1·5	1·1
Dairy products	1·5	1·1	1·4	1·2	1·5	1·1	1·5	1·1	1·5	1·1	1·5	1·1
Fish	1·5	1·1	1·5	1·1	1·5	1·1	1·5	1·1	1·5	1·1	1·5	1·1
Red and processed meat	1·5	1·1	1·5	1·1	1·5	1·1	1·5	1·1	1·5	1·1	1·5	1·1
SSB	1·9	1·2	1·7	1·2	2·0	1·2	2·0	1·2	1·7	1·2	2·0	1·2
Sodium	1·5	1·1	1·5	1·1	1·5	1·1	1·5	1·1	1·5	1·1	1·5	1·1
DASH	22·2	5·3	22·7	4·4	23·1*	3·9	22·9	4·9	23·4	4·4	23·2	4·2
Fruits	2·0	1·4	1·9	1·4	2·0	1·4	2·0	1·4	2·0	1·4	2·0	1·4
Vegetables	2·0	1·4	2·0	1·4	2·0	1·4	2·0	1·4	2·0	1·4	2·0	1·4
Whole grains	0·7	1·3	1·8**	1·5	2·0**	1·4	0·9	1·4	1·9**	1·5	2·0**	1·4
Nuts and legume	2·0	1·4	2·0	1·4	2·0	1·4	2·0	1·4	2·0	1·4	2·0	1·4
Low-fat dairy products	0·9	1·4	0·6	1·2	0·4**	1·0	1·2	1·5	0·8*	1·3	0·5**	1·2
Red and processed meat	2·0	1·4	2·0	1·4	2·0	1·4	2·0	1·4	2·0	1·4	2·0	1·4
SSB	2·6	1·5	2·4	1·5	2·7	1·5	2·7	1·5	2·7	1·4	2·7	1·5
Sodium	2·0	1·4	2·0	1·4	2·0	1·4	2·0	1·4	2·0	1·4	2·0	1·4
AMED	3·9	1·5	3·8	1·6	4·3*	1·8	3·9	1·7	3·7	1·5	4·2	1·8
Fruits	0·50	0·50	0·50	0·50	0·50	0·50	0·50	0·50	0·50	0·50	0·50	0·50
Vegetables	0·50	0·50	0·50	0·50	0·50	0·50	0·50	0·50	0·50	0·50	0·50	0·50
Whole grains	0·29	0·46	0·50**	0·50	0·50**	0·50	0·37	0·48	0·50*	0·50	0·50*	0·50
Nuts	0·39	0·49	0·00**	0·00	0·50	0·50	0·32	0·47	0·00**	0·00	0·50**	0·50
Legumes	0·50	0·50	0·50	0·50	0·50	0·50	0·50	0·50	0·50	0·50	0·50	0·50
Fish	0·50	0·50	0·50	0·50	0·50	0·50	0·50	0·50	0·50	0·50	0·50	0·50
Red and processed meat	0·50	0·50	0·50	0·50	0·50	0·50	0·50	0·50	0·50	0·50	0·50	0·50
Ratio of MUFA to SFA	0·50	0·50	0·50	0·50	0·50	0·50	0·50	0·50	0·50	0·50	0·50	0·50
Alcohol	0·18	0·39	0·23	0·43	0·23	0·42	0·22	0·41	0·14*	0·34	0·19	0·39

DQSJ, Diet Quality Score for Japanese; SSB, sugar-sweetened beverages; DASH, Dietary Approaches to Stop Hypertension; AMED, Alternate Mediterranean Diet score.

*P < 0·05, **P < 0·01, the values derived from the BDHQ and MDHQ were compared with those derived from the dietary records using a paired *t* test.

† The diet quality scores were calculated using energy-adjusted values (density method).

As shown in Table 3, Pearson’s correlation coefficients between the diet quality scores from the web questionnaires and those from the dietary records varied among the diet quality scores but did not vary between sexes or questionnaires. The range of coefficients across both sexes and both questionnaires were 0·57–0·63 for the DQSJ, 0·49–0·57 for the DASH and 0·31–0·49 for the AMED.

**Table 3.** Pearson’s correlation coefficients between the diet quality scores derived from the 4-d weighed dietary record, the web version of the Brief Self-administered Diet History Questionnaire (BDHQ) and the Meal-based Diet History Questionnaire (MDHQ) in Japanese adults^*^ (95 % CI)

	Men (*n* 111)		Women (*n* 111)	
	*r*	95 % CI	*r*	95 % CI
BDHQ				
DQSJ	0·61	0·47, 0·71	0·59	0·46, 0·70
DASH	0·56	0·41, 0·67	0·55	0·41, 0·67
AMED	0·38	0·21, 0·53	0·31	0·14, 0·47
MDHQ				
DQSJ	0·63	0·50, 0·73	0·57	0·43, 0·69
DASH	0·57	0·42, 0·68	0·49	0·33, 0·62
AMED	0·32	0·14, 0·48	0·49	0·33, 0·62

DQSJ, Diet Quality Score for Japanese; DASH, Dietary Approaches to Stop Hypertension; AMED, Alternate Mediterranean Diet score.

* The diet quality scores were calculated using energy-adjusted values (density method).

**Fig. 1.**
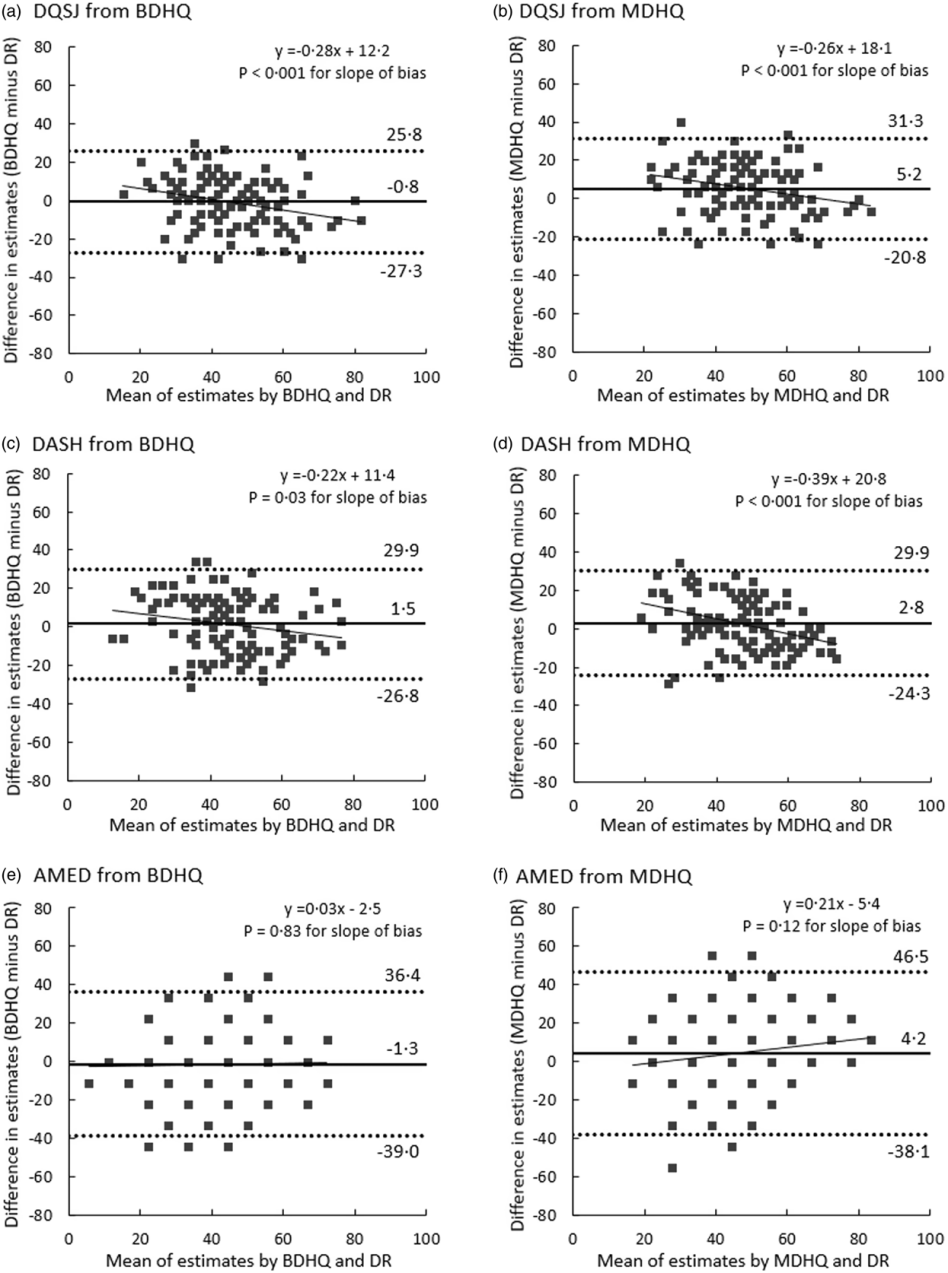
Bland–Altman plots assessing the agreement between the diet quality scores derived from the web versions of the Brief-type Self-administered Diet History Questionnaire (BDHQ) and the Meal-based Diet History Questionnaire (MDHQ) and those derived from the 4-d weighed dietary records (DR) in 111 Japanese men. Solid lines indicate mean differences and dashed lines indicate upper and lower 95 % limits of agreement. A dot may indicate two or more participants, not necessarily a single participant. The diet quality scores were calculated using energy-adjusted values (density method). AMED, Alternate Mediterranean Diet score; DASH, Dietary Approaches to Stop Hypertension; DQSJ, Diet Quality Score for Japanese.

**Fig. 2.**
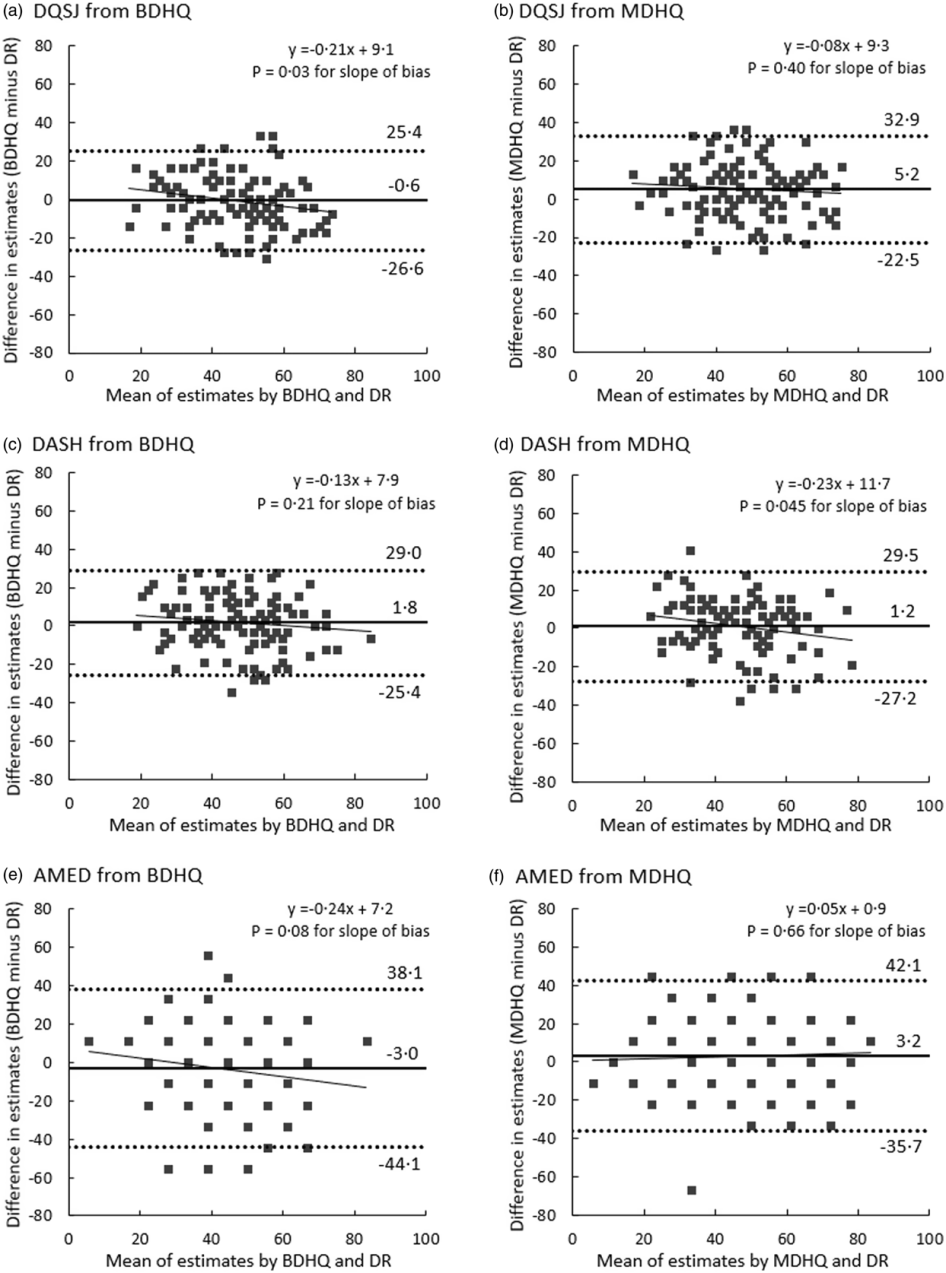
Bland–Altman plots assessing the agreement between the diet quality scores derived from the web versions of the Brief-type Self-administered Diet History Questionnaire (BDHQ) and the Meal-based Diet History Questionnaire (MDHQ) and those derived from the 4-d weighed dietary records (DR) in 111 Japanese women. Solid lines indicate mean differences and dashed lines indicate upper and lower 95 % limits of agreement. A dot may indicate two or more participants, not necessarily a single participant. The diet quality scores were calculated using energy-adjusted values (density method). AMED, Alternate Mediterranean Diet score; DASH, Dietary Approaches to Stop Hypertension; DQSJ, Diet Quality Score for Japanese.

Figures 1 and 2 show Bland–Altman plots assessing agreement between estimates of the DQSJ, DASH and AMED derived from the dietary records and those derived from the web BDHQ and web MDHQ, when calculating all scorers with possible ranges of 0–100. As the AMED has a limited number of possible scores (only ten possible scores) compared with the DQSJ and DASH, more participants were included in the same dots in the AMED than in the DQSJ and DASH. Regardless of sex or questionnaire, the limits of agreement of the DQSJ and DASH were between **–**28 % and 33 %, indicating that 95 % of participants’ estimates from the BDHQ and MDHQ were between 72 % and 133 % of their dietary records estimates. The limits of agreement for the AMED were between **–**45 % and 47 %, indicating that 95 % of participants’ estimates from the BDHQ and MDHQ were between 55 % and 147 % of their dietary records estimates. In men, the DQSJ and DASH tended to be overestimated in both questionnaires as the average score decreased, whereas the AMED did not show a proportional bias. In women, the DQSJ from the BDHQ and DASH from the MDHQ tended to be overestimated in both questionnaires as the average score decreased, but proportional bias was not observed for the other score (i.e. DQSJ from MDHQ, DASH from BDHQ and AMED from both questionnaires).

### Scores for each meal with the web version of the Meal-based Diet History Questionnaire

The number of non-consumers was six (all men) for breakfast, four (all women) for lunch, zero for dinner and ten (eight men and two women) for snacks assessed by the MDHQ and four (three men and one woman) for breakfast, one (one man) for lunch, zero for dinner and four (two men and two women) for snacks assessed by the 4-d dietary records. For breakfast, the mean scores were higher in the MDHQ than the dietary records for the DQSJ (only for women) and the AMED (online Supplementary Table 3). Similarly, for lunch and dinner, the mean values of all scores were higher in the MDHQ than in the dietary records (online Supplementary Tables 4 and 5). For snacks, the mean estimates of all the scores were lower in the MDHQ than in the dietary records in women, whereas only those of the AMED were lower in the MDHQ than the dietary records in men (online Supplementary Table 6). Pearson’s correlation coefficients of total scores derived from the MDHQ and the dietary records – combined for all three scores, both sexes and both questionnaires – varied among meals, ranging from 0·44 to 0·55 for breakfast, 0·24 to 0·47 for lunch, 0·18 to 0·36 for dinner and 0·01 to 0·31 for snacks (Table 4), with a median of 0·35. For breakfast, lunch and dinner, the limits of agreement were generally wider in the AMED than in the DQSJ and DASH, regardless of sex (online Supplementary Figures 1 and 2). Proportional bias was observed in all diet quality scores between the MDHQ and dietary records in snacks for both sexes, indicating that MDHQ tended to underestimate higher diet quality. For other meals, proportional bias was not observed for any meal in women. In men, proportional bias was observed only for the DQSJ and AMED for breakfast and the AMED for lunch.

**Table 4.** Pearson’s correlation coefficients between the diet quality scores derived from the 4-d weighed dietary record and the web version of the Meal-based Diet History Questionnaire (MDHQ), according to each meal in Japanese adults^*^ (95 % CI)

	Men (*n* 111)		Women (*n* 111)	
	r	95 % CI	r	95 % CI
Breakfast				
DQSJ	0·55	0·40, 0·66	0·47	0·31, 0·60
DASH	0·44	0·27, 0·58	0·44	0·28, 0·58
AMED	0·51	0·35, 0·63	0·52	0·38, 0·65
Lunch				
DQSJ	0·38	0·21, 0·53	0·38	0·21, 0·53
DASH	0·47	0·32, 0·61	0·40	0·24, 0·55
AMED	0·24	0·06, 0·41	0·35	0·17, 0·50
Dinner				
DQSJ	0·32	0·14, 0·48	0·34	0·16, 0·50
DASH	0·30	0·12, 0·46	0·36	0·18, 0·51
AMED	0·18	–0·01, 0·35	0·35	0·17, 0·50
Snacks				
DQSJ	0·31	0·13, 0·47	0·26	0·08, 0·43
DASH	0·20	0·01, 0·37	0·01	–0·18, 0·19
AMED	0·30	0·12, 0·46	0·22	0·03, 0·39

DQSJ, Diet Quality Score for Japanese; DASH, Dietary Approaches to Stop Hypertension; AMED, Alternate Mediterranean Diet score.

* The diet quality scores were calculated using energy-adjusted values using energy intake from the meal (density method).

### Scores for overall diet on the paper versions of the two questionnaires

The mean total scores for the DQSJ, DASH and AMED derived from the paper version of the BDHQ were not significantly different from those derived from the dietary records, except for the DASH for men (online Supplementary Table 7). Meanwhile, the mean total scores derived from the paper version of the MDHQ were significantly higher than those derived from the dietary records for the DQSJ in both sexes and for the AMED in men. Pearson’s correlation coefficients of scores derived from both questionnaires and the dietary records – combined for both sexes and both questionnaires – were 0·57–0·70 for the DQSJ, 0·49–0·66 for the DASH and 0·32–0·50 for the AMED (online Supplementary Table 8). Bland–Altman plots analysis showed similar results to those with the web versions of the questionnaires (data not shown).

## Discussion

This study examined the relative validity of the three diet quality scores (DQSJ, DASH and AMED) derived from web and paper versions of the two questionnaires (BDHQ and MDHQ) against 4-d dietary records in Japanese adults. The results for the paper versions of the BDHQ and MDHQ were generally similar to those for their web versions. To our knowledge, this is the first study to examine the validity of questionnaires for estimating easily calculable diet quality scores in Japan. This study described the scoring methods used to calculate the diet quality scores in detail, enabling researchers to calculate the diet quality scores using the BDHQ and MDHQ with standardised methods. Considering the wide use of the BDHQ, our findings can facilitate future research on the relationships between diet quality and outcomes in various Japanese datasets. Additionally, this study showed the MDHQ can be used for assessing the diet quality scores across most meal types in Japanese adults, although cautions are needed when evaluating dinner and snacks.

According to the criteria proposed in a previous review^(44)^, the web BDHQ and web MDHQ showed good ranking ability (correlation coefficients more than 0·50) for the DQSJ and DASH and acceptable ability (correlation coefficients ranged 0·20–0·49) for the AMED. Additionally, correlation coefficients observed in the present study were not inferior to those in previous studies, while direct comparison is difficult due to differences in the study population, design and scoring method. For example, in a US study, Spearman’s correlation coefficients between a questionnaire and dietary records were 0·68 in men and 0·64 in women for the DASH and 0·57 in men and 0·51 in women for AMED^(23)^. In Spanish adults, Pearson’s correlation coefficients between a questionnaire and dietary recalls were 0·33 for the modified Mediterranean diet score and 0·42 for the Mediterranean-like diet score^(18)^. In the European population, Spearman’s correlation coefficients between a questionnaire and dietary recalls were 0·32 in men and 0·56 in women for the traditional Mediterranean diet score and 0·39 in men and 0·49 in women for the Mediterranean Diet Pyramid Index^(45)^. Similar to the previous studies, correlation coefficients did not largely differ between men and women, despite the sex differences in the intake distribution used for calculating the diet quality scores. Our results showed that the BDHQ and MDHQ can be used for ranking individuals by the three diet quality scores for both men and women.

This study showed that the diet quality scores from the web BDHQ and web MDHQ for 95 % of the participants were between 70 % and 130 % of their dietary records estimates for the DQSJ and DASH and between 50 % and 150 % for the AMED. A previous study showed that the 95 % limits of agreements between a questionnaire and dietary recalls were 69 % to 134 % for a modified Mediterranean diet score^(18)^. This range was similar to the present results of the DQSJ and DASH. However, the range of the previous study was somewhat narrower than the present results of the AMED, which may be due to the wider possible score range of a modified Mediterranean diet score in the previous study (10–30) than of the AMED in the present study (0–9). Although the criteria to interpret the limit of agreement has not been established, the limits of agreement shown in the present study should be considered when estimating the diet quality scores for individuals using the BDHQ and MDHQ.

Irrespective of sex and questionnaire, the AMED showed relatively low correlation coefficients between the questionnaires and dietary records than the DQSJ and DASH. A previous US study also showed that the AMED had relatively low correlations between a questionnaire and dietary records compared with the DASH^(23)^. This may be partly explained by the limited possible score range for components (0 or 1) and the total score (0–9) of the AMED, which may lead to a lower capacity to distinguish diet quality. On the other hand, the DQSJ had somewhat high correlation coefficients between the questionnaires and dietary records compared with the other diet quality scores (DASH and AMED in the present study and HEI-2015 and NRF9.3 in previous studies^(19,20)^). As described in a previous study, the DQSJ was developed with consideration to Japanese dietary intake and showed associations with a lower prevalence of participants with inadequate intake for most nutrients examined^(13)^. Taken together, the DQSJ may be an optimal choice in assessing diet quality using the BDHQ and MDHQ in Japanese.

The ranking ability of the web MDHQ to estimate the DQSJ, DASH, and AMED was acceptable to good for breakfast, acceptable for lunch and poor to acceptable for dinner and snacks according to the criteria in a previous review^(44)^. These results were similar to the results of another study, which showed the highest correlation coefficients of other diet quality scores in breakfast and the lowest ones in snacks^(20)^. The differences in ranking ability between meal types may be attributed to the between-individual variation in the intake of foods used to calculate each component score^(36,46)^. For example, regarding snacks, several components (e.g. whole grains, fish, and red and processed meat) of the DQSJ, DASH, and AMED were not consumed by most participants and did not contribute to the variations in diet quality scores for snacks. These three scores were not originally developed for the purpose of evaluating diet quality for each meal, but rather for the habitual daily diet. To take advantage of the MDHQ’s ability to measure dietary intake for each meal, it may be necessary to develop appropriate dietary quality scores for each meal^(47)^.

Several limitations of this study warrant mention. First, although the study participants were gathered from diverse regions in Japan, they were not a nationally representative sample, but volunteers. They might therefore have been more health conscious than the general population, with limited variation in dietary intake. For example, the highest level of educational attainment in the present population was somewhat higher than that in a nationally representative sample aged 30–69 years (46 % for elementary, junior high, or high school, 11 % for junior college or technical school, and 39 % for a university degree or higher in men, 45 %, 29 % and 21 %, respectively, in women)^(48)^. However, diet quality was comparable with those in a national survey in Japan: median values of the HEI-2015 were 51·3 for women and 49·5 for men in this study population^(20)^ and mean value of the HEI-2015 was 52·2 in the national survey^(9)^. Also, the participants of the present study had similar BMI and current smoking status to the national survey^(9)^.

Second, the weighed dietary records used as a reference method in this study have measurement errors, including potential changes in habitual dietary behaviours and difficulty in capturing habitual dietary intake due to day-to-day variations in dietary intake^(39)^, which may result in underestimating the validity of the BDHQ and MDHQ. On the other hand, dietary records and dietary assessment questionnaires share the error in food composition tables, which may lead to overestimating the validity of the BDHQ and MDHQ. Nevertheless, the weighed dietary records are considered the first choice for a validation study of dietary assessment questionnaires because it is minimally dependent on memory, and its errors would be less correlated with questionnaires compared with those in dietary recall^(39)^. Additionally, the recording period of the dietary records (4 d) in this study might not have been sufficient to accurately estimate the score of food groups occasionally consumed. Nevertheless, considering the possibility that long-term dietary records may reduce participants’ motivation and alter dietary intake^(49)^, this study set a recording period of 4 d.

Finally, the sequence of administration of the BDHQ and MDHQ may affect the results of the BDHQ, since the participants completed the BDHQ after the MDHQ, which asks about the frequency of food group intake in each meal. The results for the BDHQ may not be completely independent of those of the MDHQ, which may in turn have resulted in an overestimation of the validity of the BDHQ.

In conclusion, the ability of the BDHQ and MDHQ to rank individuals in the population was good for the DQSJ and DASH and acceptable for the AMED among Japanese adults. For each meal, the ability of the MDHQ to rank individuals according to the three scores was acceptable to good for breakfast, acceptable for lunch, and poor to acceptable for dinner and snacks. The diet quality scores of most participants estimated from the BDHQ and MDHQ were between 70–130 % of estimates from dietary records for the DQSJ and DASH and 50–150 % for the AMED. The result of this study supports the validity of the BDHQ and MDHQ for use in examining the relationship between diet quality scores and outcomes in future studies of Japanese adults. However, researchers should be cautious when comparing these scores regarding outcomes because the ability to rank individuals is somewhat low in the AMED compared with the DQSJ and DASH.

## Supporting information

Oono et al. supplementary material 1 Oono et al. supplementary material

Oono et al. supplementary material 2 Oono et al. supplementary material

## Supplementary material

For supplementary material/s referred to in this article, please visit https://doi.org/10.1017/S0007114524002058
