# Supplementary material for: Relative validity of three diet quality scores derived from the Brief-type Self-administered Diet History Questionnaire and Meal-based Diet History Questionnaire in Japanese adults
Source: Br J Nutr. 2024 Nov 12;132(12):1663–73. doi: 10.1017/S0007114524002058 (PMC11695110; doi:10.1017/S0007114524002058)
Supplement: Oono et al. supplementary material 2 — Oono et al. supplementary material [file S0007114524002058sup002.pdf]

Supplemental Table 1 Criteria for the highest scores of the Diet Quality Score for Japanese (DQSJ), Dietary Approach to Stop Hypertension (DASH), and Alternate Mediterranean Diet Index (AMED), calculated from 4-day dietary records

Component	DQSJ *	DASH †	AMED ‡
Fruits	>33, >62	>48, >73	>16, >30
Vegetables	>129, >145	>161, >189	>121, >128
Whole grain §	>6, >10	>7, >15	Consumers
Dairy	>75, >115	Low-fat dairy: >39, >47	-
Nuts	>2, >4	>31, >46	Consumers
Legumes	>28, >39		>17, >21
Fish (or seafood)	>25, >28	-	>21, >22
Ratio of MUFA to SFA	-	-	>1.3, >1.3
Red and processed meat	<20, <18	<18, <16	<28, <28
SSBs	Non consumers	Non consumers	-
Sodium	<1.62, <1.59	<1.56, <1.54	-
Alcohol	-	-	5–25 g/d ethanol

PUFA, polyunsaturated fatty acids; MUFA; monounsaturated fatty acids; SFA, saturated fatty acids; SSBs, sugar-sweetened beverages; sv, serving.

Values are the intake (g/4184 kJ except for alcohol) required to achieve a maximum score of their components as assessed using 4-day dietary records in this study population (men, women).

* DQSJ ranges from possible values of 0 to 30 (1). The maximum points for each component were 3 and the minimum points were 0. Scores were calculated using sex-specific quartile intakes of components in this population. For whole grains and nuts, 0 points were assigned to non-consumers, while 1 to 3 points were assigned to consumers according to tertile intake among consumers. For SSBs, 3 points were assigned to non-consumers, while 0 to 2 points were assigned to consumers according to tertile intake among consumers. Definitions of food groups for calculation of the DQSJ were as follows: fruits did not include fruit juice and jam; vegetables included non-starchy vegetables, seaweeds, and mushrooms (not including pickles, starchy vegetables, vegetable seasoning, and vegetable juice); nuts included peanuts and other nuts; fish did not include fish roe, shellfish, octopus and squid, crustacean, and processed seafood; SSBs included lactic acid bacteria beverages, fruit juice excluding 100% fruit juice, cocoa, cola, and other sugar-sweetened soft drink (including sports drinks), tea and coffee with sugar.

† DASH ranges from possible values of 8 to 40 (2). The maximum number of points for each component was 5 and the minimum points was 1. Scores were calculated using sex-specific quintile intakes of components in this population. Because more than 20% of participants did not consume whole grain and low-fat dairy, 1 point was assigned to non-consumers, and 2–5 points were assigned to consumers by quartile of intake among consumers. Fruits included all fruits and fruit juice; vegetables did not include potatoes and legumes; and low-fat dairy included all reduced fat dairies such as milk and yogurt. Other definitions were the same as for the DQSJ.

‡ AMED ranges from possible values of 0 to 9 (3). The maximum number of points for each component was 1. The scores were calculated using the sex-specific median intakes of components in this population except for alcohol. When participant intake did not meet the criterion with 1 point, the participant received 0 points. Because more than half of the participants did not consume whole grain and nuts in this study, non-consumers received 0 points, and consumers received 1 point. Fruits included all fruits and fruit juice; vegetables did not include potatoes; and fish included fish and shrimp, and breaded fish. Other definitions were the same as in the DQSJ.

§ Values are dry weight.

Supplemental Table 2 Food items included when calculating the Diet Quality Score for Japanese (DQSJ), Dietary Approach to Stop Hypertension (DASH), and Alternate Mediterranean Diet Index (AMED) using the brief self-administered diet history questionnaire (BDHQ) and the Meal-based Diet History Questionnaire (MDHQ)

Component	Score	BDHQ	MDHQ
Fruits	DQSJ	Citrus fruit including oranges; strawberries, persimmons and kiwi fruit; other fruits	Strawberries; persimmons; citrus; kiwi fruit; watermelon; pears; bananas; grapes; melon; peaches; apples; all other fruits
	DASH, AMED	DQSJ + fruits and vegetable juice*	DQSJ + fruits and vegetable juice *
Vegetables	DQSJ	Carrots and pumpkins; tomatoes, tomato ketchup, boiled tomato and stewed tomato; green leafy vegetables including broccoli; raw vegetables used in salad (cabbage and lettuce); cabbage and Chinese cabbage; radishes and turnips; other root vegetables (onions, burdock and lotus root); mushrooms, seaweeds	Edamame (i.e., immature soybeans) and peas; seaweeds; pumpkins; mushrooms; cabbage; cucumbers; bitter melon; burdock; radishes; onions; Chinese cabbage; tomatoes; eggplants; carrots; green peppers; broccoli; green leafy vegetables; bean sprouts; lettuce; all other vegetables
	DASH, AMED	DQSJ + salted green and yellow vegetable pickles; other salted vegetable pickles (excluding salted pickled plum)	DQSJ + pickled vegetables
Whole grain	DQSJ, DASH, AMED	Buckwheat noodles; whole grain rice (brown rice, germ rice, and rice with barley) †	Brown rice; wholegrain bread; buckwheat noodles
Dairy	DQSJ	Full-fat milk and yoghurt; low-fat milk and yoghurt	Cheese; low-fat milk; yogurt; full-fat milk; all other dairy products
Low fat dairy	DASH	Low-fat milk and yoghurt	Low-fat milk
Legumes	DQSJ, AMED	Tofu (i.e. soyabean curd) and tofu products; natto (i.e. fermented soyabeans)	Soy milk; tofu (i.e., soybean curd); natto (i.e., fermented soybeans); tofu products (e.g., nama-age, abura-age, and ganmodoki)
Nuts	DQSJ, AMED	Not included	Peanuts and nuts

Legumes and nuts	DASH	Tofu (i.e. soyabean curd) and tofu products; natto (i.e. fermented soyabeans)	Soy milk; tofu (i.e., soybean curd); natto (i.e., fermented soybeans); tofu products (e.g., nama-age, abura-age, and ganmodoki); peanuts and nuts
Fish	DQSJ	Dried fish and ground fish meat products (e.g., kamaboko, chikuwa) ‡; small fish with bones; canned tuna; oily fish (including sardines, mackerel, saury, amberjack, herring, eel and fatty tuna); non-oily fish (including salmon, trout, white meat fish, freshwater fish and bonito)	Oily fish; red meat fish; eel; small fish with bones; fish eggs; dried fish; salmon; white meat fish; canned tuna
Fish and shellfish	AMED	Dried fish and ground fish meat products (e.g., kamaboko, chikuwa); small fish with bones; canned tuna; oily fish (including sardines, mackerel, saury, amberjack, herring, eel and fatty tuna); non-oily fish (including salmon, trout, white meat fish, freshwater fish and bonito); squid, octopus, shrimp and clam	Oily fish; red meat fish; squid and octopus; eel; shrimp and crab; shellfish; small fish with bones; fish eggs; dried fish; salmon; white meat fish; ground fish meat products; canned tuna; all other fish and shellfish
Red and Processed meat	DQSJ, DASH, AMED	pork and beef (including ground pork and beef); liver §; ham, sausages and bacon	Liver §; processed meat; beef; pork; all other meat
SSBs	DQSJ, DASH	Cola and sweetened soft drinks (including sports drinks)	SSB (e.g., soda, sports drinks, and sweetened fruits juice)

AMED, Alternate Mediterranean Diet score; DASH, Dietary Approaches to Stop Hypertension; DQSJ, Diet Quality Score for Japanese; SSB, sugar sweetened beverages.

* Fruits and vegetable juice was multiplied by 0.5, assuming fruits juice account for half the total amount.

† Whole grain rice was estimated by multiplying total rice intake and the corresponding values of frequency of eating whole grain rice (always, 1.0; sometimes, 0.4; rarely, 0.2; no, 0.0).

‡ "Dried fish and ground fish meat products" was multiplied by 0.5, assuming ground fish meat products account for half the total amount.

§ Liver was multiplied by 0.8, assuming chicken liver accounts for 20% of the total liver.

Supplemental Table 3 Mean estimates of the three diet quality scores for breakfast derived from the 4-day weighed dietary record (DR) and those derived from the web version of the Meal-based Diet History Questionnaire (MDHQ) in Japanese adults †

	Men (n = 111)				Women (n = 111)			
	DR		MDHQ		DR		MDHQ	
	Mean	SD	Mean	SD	Mean	SD	Mean	SD
DQSJ	11.9	3.6	12.5	3.5	12.7	3.7	14.3**	3.9
Fruits	0.9	1.1	0.9	1.1	1.1	1.2	1.2	1.2
Vegetables	1.4	1.1	1.1**	1.2	1.5	1.1	1.3	1.2
Whole grains	0.2	0.7	0.9**	1.1	0.4	0.9	1.4**	1.2
Nuts	0.4	0.9	0.9**	1.1	0.4	0.9	1.1**	1.2
Legume	1.0	1.2	1.1	1.2	1.0	1.2	1.3**	1.2
Dairy	1.2	1.2	1.0*	1.2	1.5	1.1	1.6	1.1
Fish	0.6	1.0	0.7	1.1	0.5	1.0	0.8**	1.1
Red and processed meat	1.8	1.2	1.7	1.2	2.0	1.2	1.5**	1.1
SSBs	2.8	0.7	2.7	0.7	2.9	0.5	2.7**	0.8
Sodium	1.5	1.1	1.5	1.1	1.5	1.1	1.5	1.1
DASH	21.6	3.8	21.7	3.0	22.7	4.2	22.8	3.7
Fruits	1.2	1.5	1.3	1.5	1.5	1.5	1.6	1.5
Vegetables	1.8	1.5	1.5*	1.5	1.8	1.5	1.7	1.5
Whole grains	0.3	0.9	1.2**	1.5	0.5	1.1	1.7**	1.5
Nuts and Legume	1.6	1.5	1.4	1.5	1.5	1.5	1.6	1.5
Low fat dairy	0.4	1.1	0.3	0.9	0.7	1.3	0.4*	1.1
Red and processed meat	2.5	1.5	2.3	1.5	2.7	1.5	2.1**	1.5
SSBs	3.7	0.9	3.7	0.9	3.9	0.6	3.6*	1.0
Sodium	2.0	1.4	2.0	1.4	2.0	1.4	2.0	1.4
AMED	3.2	1.7	3.8**	2.1	3.2	1.7	3.9**	1.9
Fruits	0.5	0.5	0.5	0.5	0.5	0.5	0.5	0.5
Vegetables	0.5	0.5	0.5	0.5	0.5	0.5	0.5	0.5
Whole grains	0.1	0.3	0.5**	0.5	0.2	0.4	0.5**	0.5
Nuts	0.2	0.4	0.5**	0.5	0.2	0.4	0.5**	0.5
Legumes	0.5	0.5	0.5	0.5	0.5	0.5	0.5	0.5
Fish	0.3	0.5	0.3	0.5	0.3	0.5	0.4	0.5
Red and processed meat	0.5	0.5	0.5	0.5	0.5	0.5	0.5	0.5
Ratio of MUFA to SFA	0.5	0.5	0.5	0.5	0.5	0.5	0.5	0.5
Alcohol	0.0	0.0	0.0	0.0	0.0	0.0	0.0	0.0

AMED, Alternate Mediterranean Diet score; DASH, Dietary Approaches to Stop Hypertension; DQSJ, Diet Quality Score for Japanese; MUFA; monounsaturated fatty acids; SD, standard deviation; SFA, saturated fatty acids; SSBs, sugar-sweetened beverages.
† The diet quality scores were calculated using energy adjusted values (density method).
*p<0.05, **p<0.01, the values derived from the web MDHQ questionnaires were compared with those derived from the DR using paired-t test.

Supplemental Table 4 Mean estimates of the diet quality scores for lunch derived from the 4-day weighed dietary record (DR) and those derived from the web version of the Meal-based Diet History Questionnaire (MDHQ) in Japanese adults [†]

	Men (n = 111)				Women (n = 111)			
	DR		MDHQ		DR		MDHQ	
	Mean	SD	Mean	SD	Mean	SD	Mean	SD
DQSJ	11.4	4.2	13.3**	4.2	12.2	3.8	14.3**	4.5
Fruits	0.7	1.1	0.7	1.1	1.1	1.2	1.0	1.2
Vegetables	1.5	1.1	1.5	1.1	1.5	1.1	1.6	1.1
Whole grains	0.4	0.9	1.5**	1.1	0.4	0.9	1.5**	1.1
Nuts	0.2	0.6	0.9**	1.1	0.2	0.6	1.0**	1.2
Legume	0.9	1.1	1.1	1.2	1.0	1.2	1.3*	1.2
Dairy	1.1	1.2	0.7**	1.1	1.2	1.2	1.1	1.2
Fish	1.0	1.2	1.4*	1.2	1.1	1.2	1.3	1.2
Red and processed meat	1.5	1.1	1.5	1.1	1.5	1.1	1.5	1.1
SSBs	2.7	0.8	2.6	0.9	2.7	0.8	2.6	0.9
Sodium	1.5	1.1	1.5	1.1	1.5	1.1	1.5	1.1
DASH	21.1	4.4	22.1**	3.9	21.9	4.0	22.8*	3.9
Fruits	1.0	1.4	1.0	1.4	1.5	1.5	1.3	1.5
Vegetables	2.0	1.4	2.0	1.4	2.0	1.4	2.0	1.4
Whole grains	0.5	1.1	2.0**	1.4	0.5	1.1	2.0**	1.4
Nuts and Legume	1.6	1.5	1.4	1.5	1.8	1.5	1.6	1.5
Low fat dairy	0.3	0.9	0.2	0.8	0.4	1.0	0.3	0.9
Red and processed meat	2.0	1.4	2.0	1.4	2.0	1.4	2.0	1.4
SSBs	3.6	1.0	3.5	1.1	3.6	1.0	3.5	1.1
Sodium	2.0	1.4	2.0	1.4	2.0	1.4	2.0	1.4
AMED	3.2	1.5	3.9**	2.0	3.3	1.5	4.0**	1.8
Fruits	0.4	0.5	0.4	0.5	0.5	0.5	0.5	0.5
Vegetables	0.5	0.5	0.5	0.5	0.5	0.5	0.5	0.5
Whole grains	0.2	0.4	0.5**	0.5	0.2	0.4	0.5**	0.5
Nuts	0.1	0.3	0.4**	0.5	0.1	0.3	0.5**	0.5
Legumes	0.4	0.5	0.5	0.5	0.5	0.5	0.5	0.5
Fish	0.5	0.5	0.5	0.5	0.5	0.5	0.5	0.5
Red and processed meat	0.5	0.5	0.5	0.5	0.5	0.5	0.5	0.5
Ratio of MUFA to SFA	0.5	0.5	0.5	0.5	0.5	0.5	0.5	0.5
Alcohol	0.0	0.2	0.0	0.2	0.0	0.0	0.0	0.1

AMED, Alternate Mediterranean Diet score; DASH, Dietary Approaches to Stop Hypertension; DQSJ, Diet Quality Score for Japanese; MUFA; monounsaturated fatty acids; SD, standard deviation; SFA, saturated fatty acids; SSBs, sugar-sweetened beverages.

[†] The diet quality scores were calculated using energy adjusted values (density method).

*$p<0.05$, **$p<0.01$, the values derived from the web MDHQ questionnaires were compared with those derived from the DR using paired-t test.

Supplemental Table 5 Mean estimates of the diet quality scores for dinner derived from the 4-day weighed dietary record (DR) and those derived from the web version of the Meal-based Diet History Questionnaire (MDHQ) in Japanese adults †

	Men (n = 111)				Women (n = 111)			
	DR		MDHQ		DR		MDHQ	
	Mean	SD	Mean	SD	Mean	SD	Mean	SD
DQSJ	13.0	3.5	15.0**	3.4	13.1	3.3	15.8**	3.7
Fruits	1.0	1.2	1.0	1.2	1.0	1.2	1.4**	1.2
Vegetables	1.5	1.1	1.5	1.1	1.5	1.1	1.5	1.1
Whole grains	0.2	0.7	1.5**	1.1	0.3	0.8	1.5**	1.1
Nuts	0.2	0.7	1.3**	1.2	0.1	0.5	1.5**	1.1
Legume	1.5	1.1	1.5	1.1	1.5	1.1	1.5	1.1
Dairy	1.2	1.2	0.9*	1.1	1.2	1.2	1.2	1.2
Fish	1.5	1.1	1.5	1.1	1.5	1.1	1.5	1.1
Red and processed meat	1.5	1.1	1.5	1.1	1.5	1.1	1.5	1.1
SSBs	2.9	0.5	2.7*	0.8	2.9	0.5	2.7*	0.8
Sodium	1.5	1.1	1.5	1.1	1.5	1.1	1.5	1.1
DASH	21.8	3.5	23.2**	3.1	21.9	3.4	23.6**	3.6
Fruits	1.4	1.5	1.4	1.5	1.4	1.5	1.7	1.5
Vegetables	2.0	1.4	2.0	1.4	2.0	1.4	2.0	1.4
Whole grains	0.3	0.9	2.0**	1.4	0.4	1.0	2.0**	1.4
Nuts and Legume	2.0	1.4	2.0	1.4	2.0	1.4	2.0	1.4
Low fat dairy	0.3	0.9	0.2	0.8	0.3	0.9	0.2	0.8
Red and processed meat	2.0	1.4	2.0	1.4	2.0	1.4	2.0	1.4
SSBs	3.9	0.6	3.6*	1.0	3.9	0.6	3.6*	1.0
Sodium	2.0	1.4	2.0	1.4	2.0	1.4	2.0	1.4
AMED	3.4	1.5	4.2**	1.8	3.4	1.5	4.2**	1.7
Fruits	0.5	0.5	0.5	0.5	0.5	0.5	0.5	0.5
Vegetables	0.5	0.5	0.5	0.5	0.5	0.5	0.5	0.5
Whole grains	0.1	0.3	0.5**	0.5	0.1	0.4	0.5**	0.5
Nuts	0.1	0.3	0.5**	0.5	0.0	0.2	0.5**	0.5
Legumes	0.5	0.5	0.5	0.5	0.5	0.5	0.5	0.5
Fish	0.5	0.5	0.5	0.5	0.5	0.5	0.5	0.5
Red and processed meat	0.5	0.5	0.5	0.5	0.5	0.5	0.5	0.5
Ratio of MUFA to SFA	0.5	0.5	0.5	0.5	0.5	0.5	0.5	0.5
Alcohol	0.2	0.4	0.2	0.4	0.2	0.4	0.2	0.4

AMED, Alternate Mediterranean Diet score; DASH, Dietary Approaches to Stop Hypertension; DQSJ, Diet Quality Score for Japanese; MUFA; monounsaturated fatty acids; SD, standard deviation; SFA, saturated fatty acids; SSBs, sugar-sweetened beverages.

† The diet quality scores were calculated using energy adjusted values (density method).

*$p<0.05$, **$p<0.01$, the values derived from the web MDHQ questionnaires were compared with those derived from the DR using paired-t test.

Supplemental Table 6 Mean estimates of the diet quality scores for snacks derived from the 4-day weighed dietary record (DR) and those derived from the web version of the Meal-based Diet History Questionnaire (MDHQ) in Japanese adults †

	Men (n = 111)				Women (n = 111)			
	DR		MDHQ		DR		MDHQ	
	Mean	SD	Mean	SD	Mean	SD	Mean	SD
DQSJ	8.4	2.9	8.2	2.2	9.8	3.1	8.6**	2.2
Fruits	0.4	0.9	0.8**	1.1	0.9	1.1	1.0	1.2
Vegetables	0.3	0.8	0.0**	0.0	0.4	0.9	0.0**	0.0
Whole grains	0.0	0.0	0.0	0.0	0.0	0.2	0.0	0.0
Nuts	0.2	0.7	0.0**	0.0	0.3	0.8	0.0**	0.0
Legume	0.2	0.7	0.0**	0.0	0.4	0.9	0.0**	0.0
Dairy	0.8	1.1	0.8	1.1	1.3	1.3	1.1	1.2
Fish	0.1	0.4	0.0	0.0	0.1	0.5	0.0*	0.0
Red and processed meat	2.8	0.6	3.0**	0.0	2.9	0.5	3.0*	0.0
SSBs	2.0	1.2	2.1	1.1	2.1	1.1	2.0	1.2
Sodium	1.5	1.1	1.5	1.1	1.5	1.1	1.5	1.1
DASH	18.7	3.5	18.2	2.3	19.8	3.4	18.4**	2.4
Fruits	0.7	1.3	1.1*	1.4	1.3	1.5	1.4	1.5
Vegetables	0.7	1.3	0.0**	0.0	0.8	1.3	0.0**	0.0
Whole grains	0.0	0.0	0.0	0.0	0.0	0.2	0.0	0.0
Nuts and Legume	0.5	1.2	0.0**	0.0	0.7	1.3	0.0**	0.0
Low fat dairy	0.1	0.6	0.2	0.8	0.3	0.9	0.3	0.9
Red and processed meat	3.8	0.7	4.0**	0.0	3.8	0.7	4.0*	0.0
SSBs	2.8	1.5	2.9	1.5	2.9	1.4	2.7	1.5
Sodium	2.0	1.4	2.0	1.4	2.0	1.4	2.0	1.4
AMED	2.4	1.3	2.1*	0.8	2.7	1.4	2.1**	0.7
Fruits	0.3	0.5	0.4**	0.5	0.5	0.5	0.5	0.5
Vegetables	0.3	0.5	0.0**	0.0	0.3	0.5	0.0**	0.0
Whole grains	0.0	0.0	0.0	0.0	0.0	0.1	0.0	0.0
Nuts	0.1	0.3	0.0**	0.0	0.1	0.3	0.0**	0.0
Legumes	0.1	0.3	0.0**	0.0	0.2	0.4	0.0**	0.0
Fish	0.1	0.3	0.0**	0.0	0.1	0.3	0.0**	0.0
Red and processed meat	0.9	0.3	1.0**	0.0	0.9	0.2	1.0**	0.0
Ratio of MUFA to SFA	0.5	0.5	0.5	0.5	0.5	0.5	0.5	0.5
Alcohol	0.1	0.3	0.1	0.3	0.0	0.2	0.1	0.2

AMED, Alternate Mediterranean Diet score; DASH, Dietary Approaches to Stop Hypertension; DQSJ, Diet Quality Score for Japanese; MUFA; monounsaturated fatty acids; SD, standard deviation; SFA, saturated fatty acids; SSBs, sugar-sweetened beverages.

† The diet quality scores were calculated using energy adjusted values (density method).

*p<0.05, **p<0.01, the values derived from the web MDHQ questionnaires were compared with those derived from the DR using paired-t test.

Supplemental Table 7 Mean estimates of the diet quality scores derived from the 4-day weighed dietary record (DR), the paper version of the brief self-administered diet history questionnaire (BDHQ), and the paper version of the Meal-based Diet History Questionnaire (MDHQ) in Japanese adults †

	Men (n = 111)						Women (n = 111)					
	DR		BDHQ		MDHQ		DR		BDHQ		MDHQ	
	Mean	SD	Mean	SD	Mean	SD	Mean	SD	Mean	SD	Mean	SD
DQSJ	13.8	5.0	13.6	4.0	15.4**	4.0	13.9	4.7	13.7	4.0	15.5**	4.4
Fruits	1.5	1.1	1.5	1.2	1.5	1.1	1.5	1.1	1.5	1.1	1.5	1.1
Vegetables	1.5	1.1	1.5	1.1	1.5	1.1	1.5	1.1	1.5	1.1	1.5	1.1
Whole grains	0.6	1.0	1.5**	1.1	1.5**	1.1	0.7	1.1	1.5**	1.1	1.5**	1.1
Nuts	0.8	1.1	0.0**	0.0	1.3**	1.2	0.6	1.1	0.0**	0.0	1.5**	1.1
Legume	1.5	1.1	1.5	1.1	1.5	1.1	1.5	1.1	1.5	1.1	1.5	1.1
Dairy	1.5	1.1	1.4	1.2	1.5	1.1	1.5	1.1	1.5	1.1	1.5	1.1
Fish	1.5	1.1	1.5	1.1	1.5	1.1	1.5	1.1	1.5	1.1	1.5	1.1
Red and processed meat	1.5	1.1	1.5	1.1	1.5	1.1	1.5	1.1	1.5	1.1	1.5	1.1
SSBs	1.9	1.2	1.7	1.2	2.0	1.2	2.0	1.2	1.7	1.2	2.0	1.2
Sodium	1.5	1.1	1.5	1.1	1.5	1.1	1.5	1.1	1.5	1.1	1.5	1.1
DASH	22.2	5.3	22.7	4.4	23.1*	3.9	22.9	4.9	23.4	4.4	23.2	4.2
Fruits	2.0	1.4	1.9	1.4	2.0	1.4	2.0	1.4	2.0	1.4	2.0	1.4
Vegetables	2.0	1.4	2.0	1.4	2.0	1.4	2.0	1.4	2.0	1.4	2.0	1.4
Whole grains	0.7	1.3	1.8**	1.5	2.0**	1.4	0.9	1.4	1.9**	1.5	2.0**	1.4
Nuts and Legume	2.0	1.4	2.0	1.4	2.0	1.4	2.0	1.4	2.0	1.4	2.0	1.4
Low fat dairy	0.9	1.4	0.6	1.2	0.4**	1.0	1.2	1.5	0.8*	1.3	0.5**	1.2
Red and processed meat	2.0	1.4	2.0	1.4	2.0	1.4	2.0	1.4	2.0	1.4	2.0	1.4
SSBs	2.6	1.5	2.4	1.5	2.7	1.5	2.7	1.5	2.7	1.4	2.7	1.5
Sodium	2.0	1.4	2.0	1.4	2.0	1.4	2.0	1.4	2.0	1.4	2.0	1.4
AMED	3.9	1.5	3.8	1.6	4.3*	1.8	3.9	1.7	3.7	1.5	4.2	1.8
Fruits	0.50	0.50	0.50	0.50	0.50	0.50	0.50	0.50	0.50	0.50	0.50	0.50
Vegetables	0.50	0.50	0.50	0.50	0.50	0.50	0.50	0.50	0.50	0.50	0.50	0.50

Whole grains	0.29	0.46	0.50**	0.50	0.50**	0.50	0.37	0.48	0.50*	0.50	0.50*	0.50
Nuts	0.39	0.49	0.0**	0.00	0.50	0.50	0.32	0.47	0.00**	0.00	0.50**	0.50
Legumes	0.50	0.50	0.50	0.50	0.50	0.50	0.50	0.50	0.50	0.50	0.50	0.50
Fish	0.50	0.50	0.50	0.50	0.50	0.50	0.50	0.50	0.50	0.50	0.50	0.50
Red and processed meat	0.50	0.50	0.50	0.50	0.50	0.50	0.50	0.50	0.50	0.50	0.50	0.50
Ratio of MUFA to SFA	0.50	0.50	0.50	0.50	0.50	0.50	0.50	0.50	0.50	0.50	0.50	0.50
Alcohol	0.18	0.39	0.23	0.43	0.23	0.42	0.22	0.41	0.14*	0.34	0.19	0.39

AMED, Alternate Mediterranean Diet score; DASH, Dietary Approaches to Stop Hypertension; DQSJ, Diet Quality Score for Japanese; MUFA; monounsaturated fatty acids; SD, standard deviation; SFA, saturated fatty acids; SSBs, sugar-sweetened beverages.

† The diet quality scores derived from BDHQ, MDHQ, and DR were calculated using energy adjusted values (density method).

$^{*}p<0.05$, $^{**}p<0.01$, the values derived from the web MDHQ questionnaires were compared with those derived from the DR using paired-t test.

Supplemental Table 8 Pearson correlation coefficients between the diet quality scores derived from the 4-day weighed dietary record (DR), the paper version of the brief self-administered diet history questionnaire (BDHQ), and the paper version of the Meal-based Diet History Questionnaire (MDHQ) in Japanese adults *

	Men (n = 111)		Women (n = 111)	
	r	95%CI	r	95%CI
BDHQ				
DQSJ	0.70	(0.59, 0.78)	0.57	(0.42, 0.68)
DASH	0.66	(0.53, 0.75)	0.49	(0.33, 0.62)
AMED	0.32	(0.14, 0.48)	0.33	(0.16, 0.49)
MDHQ				
DQSJ	0.68	(0.56, 0.77)	0.69	(0.58, 0.77)
DASH	0.63	(0.50, 0.73)	0.61	(0.47, 0.71)
AMED	0.39	(0.22, 0.54)	0.50	(0.34, 0.62)

AMED, Alternate Mediterranean Diet score; CI, confidence interval; DASH, Dietary Approaches to Stop Hypertension; DQSJ, Diet Quality Score for Japanese.

* The diet quality scores derived from BDHQ, MDHQ, and DR were calculated using energy adjusted values (density method).

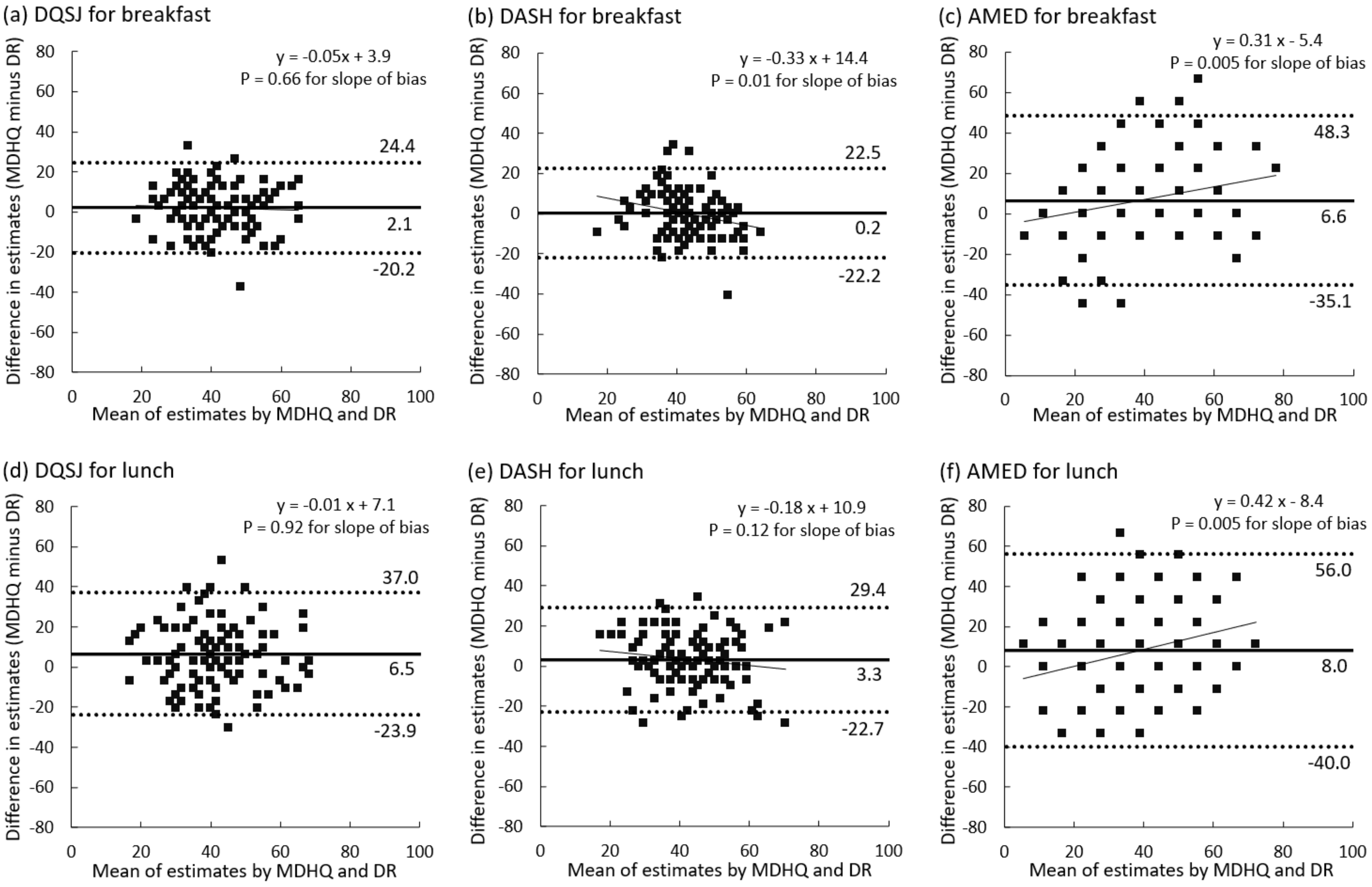
(a) DQSJ for breakfast
y = -0.05x + 3.9
P = 0.66 for slope of bias
24.4
2.1
-20.2
(b) DASH for breakfast
y = -0.33 x + 14.4
P = 0.01 for slope of bias
22.5
0.2
-22.2
(c) AMED for breakfast
y = 0.31 x - 5.4
P = 0.005 for slope of bias
48.3
6.6
-35.1
(d) DQSJ for lunch
y = -0.01 x + 7.1
P = 0.92 for slope of bias
37.0
6.5
-23.9
(e) DASH for lunch
y = -0.18 x + 10.9
P = 0.12 for slope of bias
29.4
3.3
-22.7
(f) AMED for lunch
y = 0.42 x - 8.4
P = 0.005 for slope of bias
56.0
8.0
-40.0
Difference in estimates (MDHQ minus DR)
Mean of estimates by MDHQ and DR

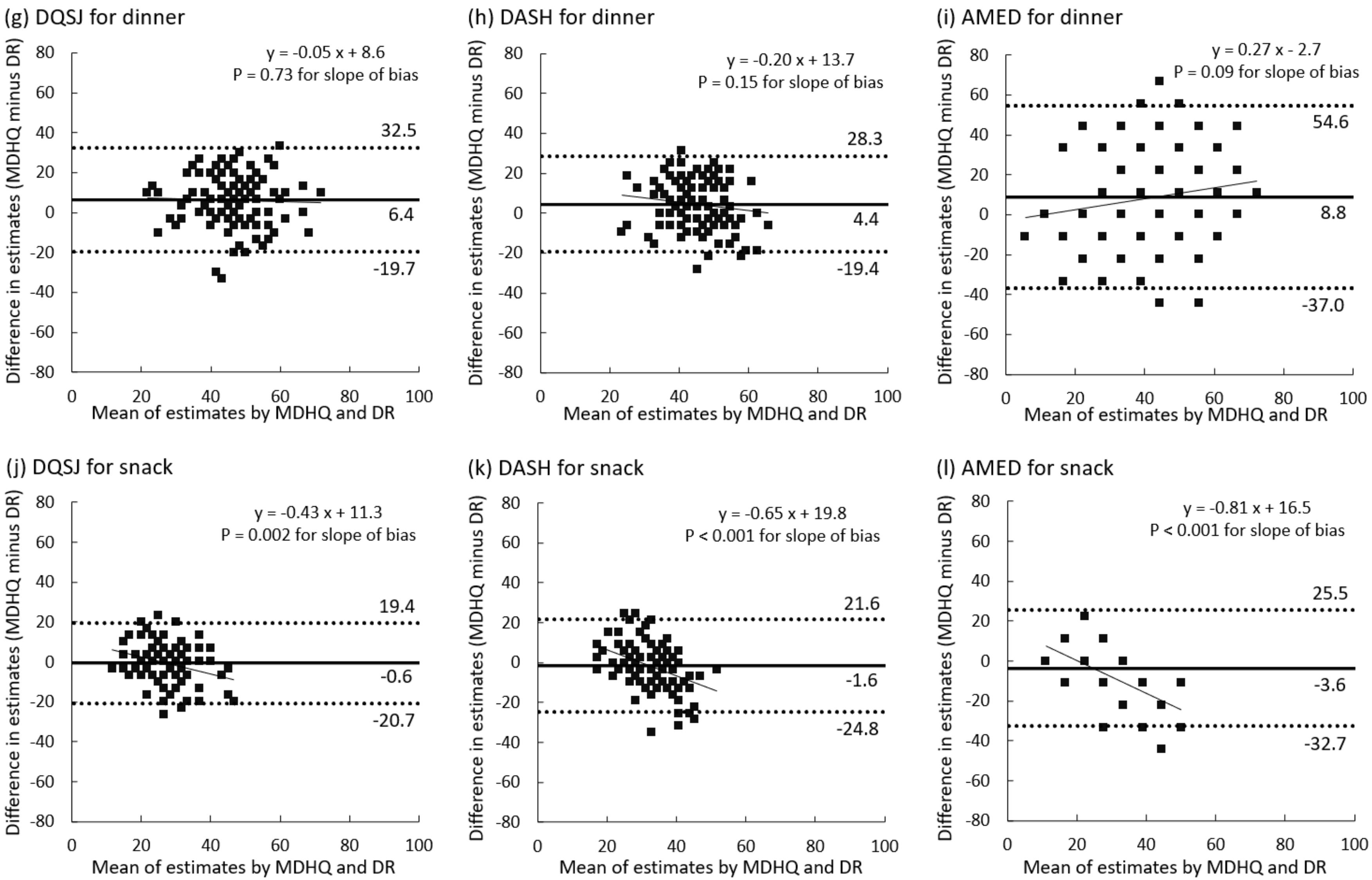
(g) DQSJ for dinner
y = -0.05 x + 8.6
P = 0.73 for slope of bias
32.5
6.4
-19.7
(h) DASH for dinner
y = -0.20 x + 13.7
P = 0.15 for slope of bias
28.3
4.4
-19.4
(i) AMED for dinner
y = 0.27 x - 2.7
P = 0.09 for slope of bias
54.6
8.8
-37.0
(j) DQSJ for snack
y = -0.43 x + 11.3
P = 0.002 for slope of bias
19.4
-0.6
-20.7
(k) DASH for snack
y = -0.65 x + 19.8
P < 0.001 for slope of bias
21.6
-1.6
-24.8
(l) AMED for snack
y = -0.81 x + 16.5
P < 0.001 for slope of bias
25.5
-3.6
-32.7
Difference in estimates (MDHQ minus DR)
Mean of estimates by MDHQ and DR
80
60
40
20
0
-20
-40
-60
-80
0
20
40
60
80
100

Supplemental Figure 1 Bland-Altman plots assessing the agreement between diet quality scores for each meal derived from the web version of the Meal-based Diet History Questionnaire (MDHQ) and those derived from the 4-day weighed dietary records (DR) in 111 Japanese men

Solid lines indicate mean differences and dashed lines indicate upper and lower 95% limits of agreement. A point may indicate two or more participants, not necessarily a participant.

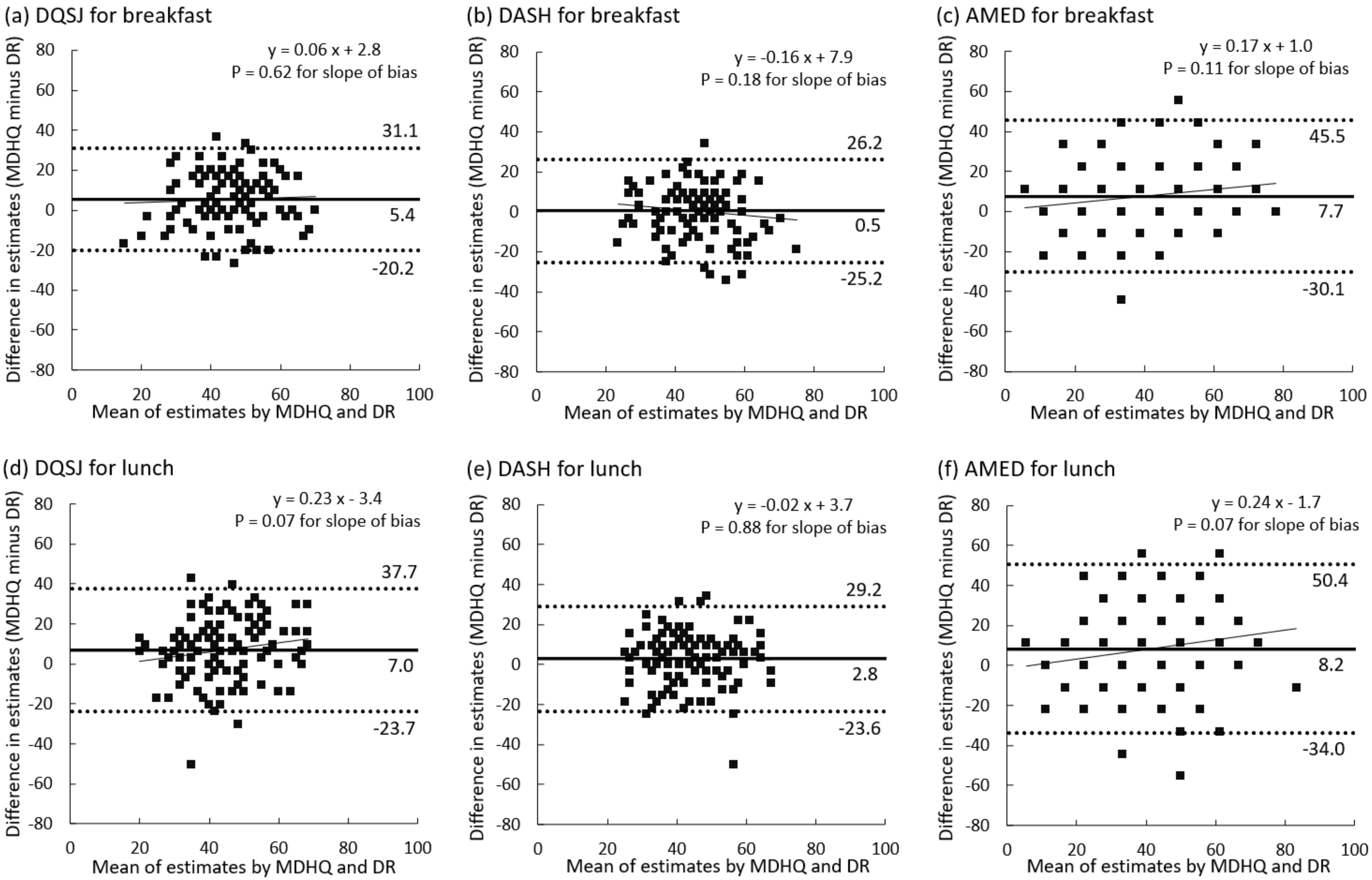
(a) DQSJ for breakfast
y = 0.06 x + 2.8
P = 0.62 for slope of bias
31.1
5.4
-20.2
(b) DASH for breakfast
y = -0.16 x + 7.9
P = 0.18 for slope of bias
26.2
0.5
-25.2
(c) AMED for breakfast
y = 0.17 x + 1.0
P = 0.11 for slope of bias
45.5
7.7
-30.1
(d) DQSJ for lunch
y = 0.23 x - 3.4
P = 0.07 for slope of bias
37.7
7.0
-23.7
(e) DASH for lunch
y = -0.02 x + 3.7
P = 0.88 for slope of bias
29.2
2.8
-23.6
(f) AMED for lunch
y = 0.24 x - 1.7
P = 0.07 for slope of bias
50.4
8.2
-34.0
Difference in estimates (MDHQ minus DR)
Mean of estimates by MDHQ and DR

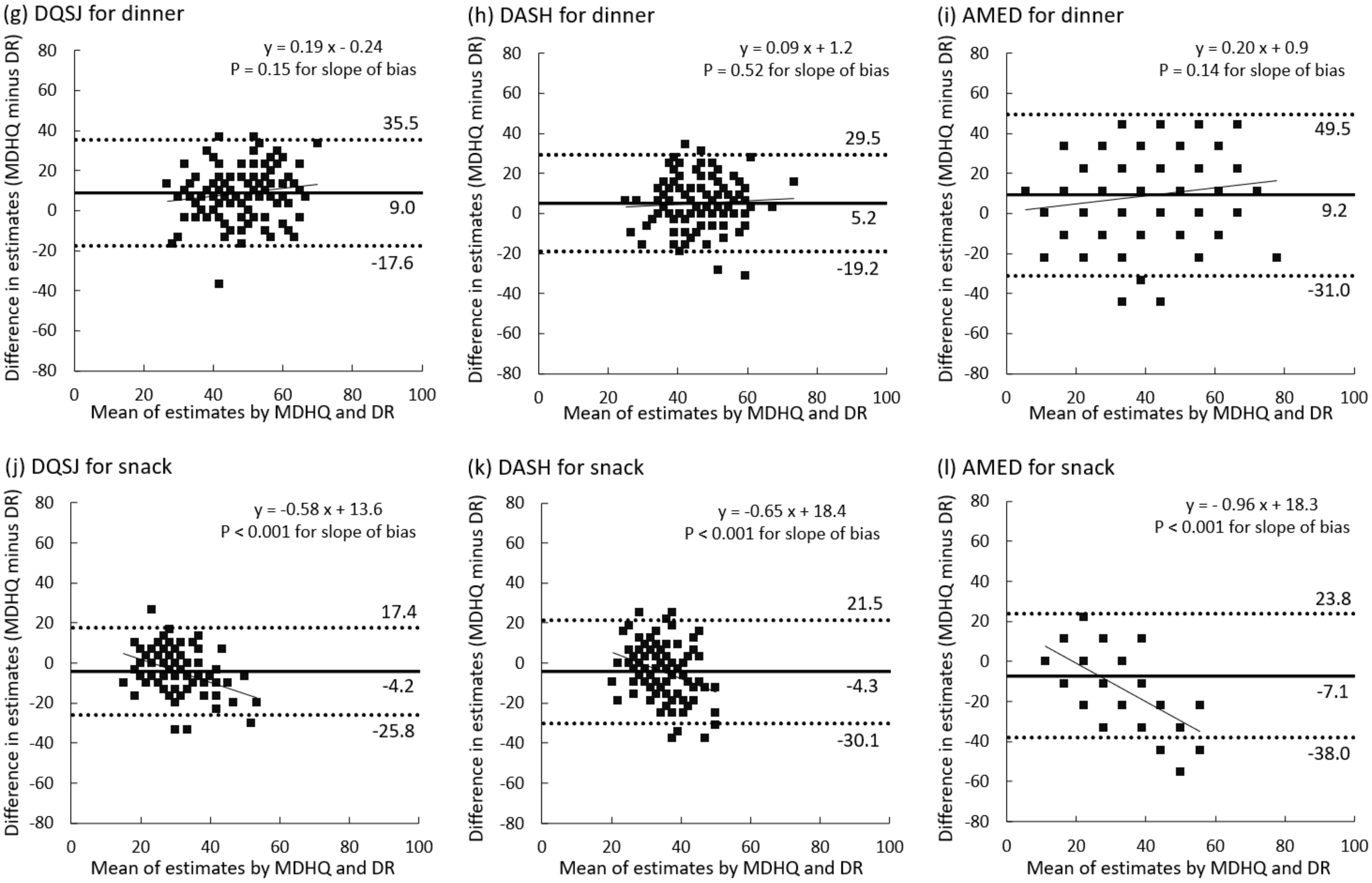
(g) DQSJ for dinner
y = 0.19 x - 0.24
P = 0.15 for slope of bias
35.5
9.0
-17.6
(h) DASH for dinner
y = 0.09 x + 1.2
P = 0.52 for slope of bias
29.5
5.2
-19.2
(i) AMED for dinner
y = 0.20 x + 0.9
P = 0.14 for slope of bias
49.5
9.2
-31.0
(j) DQSJ for snack
y = -0.58 x + 13.6
P < 0.001 for slope of bias
17.4
-4.2
-25.8
(k) DASH for snack
y = -0.65 x + 18.4
P < 0.001 for slope of bias
21.5
-4.3
-30.1
(l) AMED for snack
y = - 0.96 x + 18.3
P < 0.001 for slope of bias
23.8
-7.1
-38.0
Difference in estimates (MDHQ minus DR)
Mean of estimates by MDHQ and DR

Supplemental Figure 2 Bland-Altman plots assessing the agreement between diet quality scores for each meal derived from the web version of the Meal-based Diet History Questionnaire (MDHQ) and those derived from the 4-day weighed dietary records (DR) in 111 Japanese women
Solid lines indicate mean differences and dashed lines indicate upper and lower 95% limits of agreement. A point may indicate two or more participants, not necessarily a participant.

References

1. Oono F, Murakami K, Fujiwara A, Shinozaki N, Adachi R, Asakura K, et al. Development of a Diet Quality Score for Japanese and Comparison With Existing Diet Quality Scores Regarding Inadequacy of Nutrient Intake. J Nutr. 2023;153(3):798-810. doi: 10.1016/j.tjnut.2022.11.022.
2. Fung TT, Chiuve SE, McCullough ML, Rexrode KM, Logroscino G, Hu FB. Adherence to a DASH-style diet and risk of coronary heart disease and stroke in women. Arch Intern Med. 2008;168(7):713-20. doi: 10.1001/archinte.168.7.713.
3. Chiuve SE, Fung TT, Rimm EB, Hu FB, McCullough ML, Wang M, et al. Alternative dietary indices both strongly predict risk of chronic disease. J Nutr. 2012;142(6):1009-18. doi: 10.3945/jn.111.157222.
